# Plasma metabolomics reveals a diagnostic metabolic fingerprint for mitochondrial aconitase (ACO2) deficiency

**DOI:** 10.1371/journal.pone.0176363

**Published:** 2017-05-02

**Authors:** Lucia Abela, Ronen Spiegel, Lisa M. Crowther, Andrea Klein, Katharina Steindl, Sorina Mihaela Papuc, Pascal Joset, Yoav Zehavi, Anita Rauch, Barbara Plecko, Thomas Luke Simmons

**Affiliations:** 1 Division of Child Neurology, University Children’s Hospital Zurich, Zurich, Switzerland; 2 Children’s Research Centre, University Children’s Hospital Zurich, Zurich, Switzerland; 3 Radiz–Rare Disease Initiative Zurich, Clinical Research Priority Program for Rare Diseases, University of Zurich, Zurich, Switzerland; 4 Department of Pediatrics B, Emek Medical Center, Afula, Rappaport Faculty of Medicine, Technion, Israel; 5 Institute of Medical Genetics, University of Zurich, Schlieren, Switzerland; Mayo Clinic Rochester, UNITED STATES

## Abstract

Mitochondrial respiratory chain dysfunction has been identified in a number of neurodegenerative disorders. Infantile cerebellar-retinal degeneration associated with mutations in the mitochondrial aconitase 2 gene (*ACO2*) has been recently described as a neurodegenerative disease of autosomal recessive inheritance. To date there is no biomarker for *ACO2* deficiency and diagnosis relies on genetic analysis. Here we report global metabolic profiling in eight patients with *ACO2* deficiency. Using an LC-MS-based metabolomics platform we have identified several metabolites with affected plasma concentrations including the tricarboxylic acid cycle metabolites cis-aconitate, isocitrate and alpha-ketoglutarate, as well as phosphoenolpyruvate and hydroxybutyrate. Taken together we report a diagnostic metabolic fingerprint for mitochondrial aconitase 2 deficiency.

## Introduction

The Krebs cycle (i.e. citric acid cycle; TCA cycle) is a nexus of carbohydrate, fat and protein metabolism. Eight enzymatic reactions comprise the core TCA catalytic cycle (S1 Fig), which ultimately oxidizes acetate (acetyl-CoA) into carbon dioxide and water. The cycle converts nicotinamide adenine dinucleotide (NAD+) into reduced NAD+ (NADH), flavin adenine dinucleotide (FAD) into FADH_2_, and guanosine diphosphate (GDP) and inorganic phosphate (P_i_) into guanosine triphosphate (GTP). The NADH and FADH_2_ generated in the TCA cycle are subsequently used by the oxidative phosphorylation pathway to generate energy-rich adenosine triphosphate (ATP), which constitutes the main source of cellular energy thereby sustaining mitochondrial respiratory chain activity [1]. In eukaryotes, the citric acid cycle occurs in the mitochondrial matrix, and is of critical importance to all organisms utilizing oxygen for cellular respiration.

ACO2 is an iron-sulphur (4Fe-4S) cluster protein that catalyzes the reversible isomerization of citrate to isocitrate within the mitochondrial citric acid cycle. In addition to supplying the mitochondrial respiratory chain through the generation of the reducing molecules NADH and FADH_2_, the citric acid cycle also generates precursors for the biosynthesis of several endogenous compounds, e.g. in fatty acid synthesis and gluconeogenesis.

Infantile cerebellar-retinal degeneration associated with mutations in the mitochondrial aconitase 2 gene (*ACO2*) has been recently described in eight individuals from two unrelated Arab families [2] and three patients of French or Algerian descent [3]. Patients share a similar clinical phenotype with ophthalmological abnormalities including optic atrophy, retinal degeneration and strabismus, axial hypotonia, ataxia, progressive microcephaly and various types of seizures often triggered by infection. The disease follows a neurodegenerative course but survival into adulthood is possible. Cerebral imaging reveals progressive brain atrophy affecting primarily the cerebellum as well as thinning of the corpus callosum, demyelination and cortical atrophy of the frontal and temporal lobes. Interestingly two patients with *ACO2* mutations, reported by Metodiev *et al*., had a different phenotype with isolated optic atrophy with onset in early childhood [3].

With respect to biomarkers, severe metabolic acidosis and hyperglycemia was described in one of the two patients reported by Metodiev *et al* [3], while all patients reported by Spiegel *et al*. had normal serum and CSF lactate, pyruvate, alanine and organic acids in urine as well as a normal levels of mitochondrial respiratory chain enzymes and pyruvate dehydrogenase activity; there was only slight reduction in the oxidation of glutamate [2]. Thus *ACO2* deficiency lacks typical mitochondrial disease biomarkers, similar to other citric acid cycle defects such as succinate dehydrogenase deficiency [4], succinyl-CoA synthase deficiency [5] and fumarate hydratase deficiency [6, 7].

The diagnosis of *ACO2* deficiency relies on genetic testing or enzyme activity assay [8]. A total of seven missense mutations (p.Gly259Asp, p.Ser112Arg, p.Leu74Val, p.Gly661Arg, p.Lys736Asn, p.Pro712Lys, and p.Arg607Cit) and one frameshift mutation (p.Lys776Asnfs*49) have been reported [2, 3, 8]. All nucleotide changes involve highly conserved positions, which are predicted to be deleterious. However, the p.Leu74Val variant has been reported in the SNP database (rs141772938; MAF 0.003) [3].

Given the unspecific encephalomyopathic presentation of ACO2 deficiency and lack of a specific disease biomarker, we were interested in identifying novel disease-associated metabolites or metabolite profiles by clinical metabolomics. Mass-spectrometry based metabolomics has led to the discovery of novel biomarkers and disease-associated metabolic profiles in as yet uncharacterized genetic diseases [9, 10]. Here we performed comparative untargeted metabolomics on the plasma of eight *ACO2* deficient patients of four unrelated families against a matched control cohort and report a diagnostic metabolic fingerprint in plasma for mitochondrial aconitase 2 deficiency.

## Materials and methods

### Ethics statement

Parents or the legal guardians of all patients have given full written informed consent for participation in a study on metabolic-genetic research into early onset epileptic encephalopathies (EE) and a metabolome study, respectively. Parents or the legal guardians of the control cohort have given full written informed consent for participation in the metabolome study. Both studies have been approved by the Institutional Review Board of the Kanton of Zurich. All procedures followed were in accordance with the ethical standards of the responsible local Ethics committee (institutional and national) and with the Helsinki Declaration of 1975, as revised in 2000.

### Informed consent

Parents or the legal guardians of all patients have given full written informed consent for participation in the study.

### Patients

Eight patients from four different families (F1-P1/P2, F2-P1/P2/P3, F3-P1/P2, F4-P1) were included in this study with an age range from 22 months to 21 years (Table 1). Three families (F1-P1/P2, F2-P1/P2/P3, F3-P1/P2) were of Arab origin, one family of Caucasian origin (F4-P1). F1-P1/P2 and F2-P1/P2/P3 have been previously published [2]. Disease onset was between 2 and 6 months. The clinical phenotype was mainly characterized by severe muscular hypotonia, seizures, strabismus, optic atrophy, ataxia, profound developmental delay and secondary microcephaly. Table 1 provides a summary of the clinical, biochemical, imaging and genetic findings. The control cohort comprised 30 plasma samples (age range 1.8–17.0; mean 12.15 ±3.58, median 12.25 years) admitted to the Children’s Hospital for unrelated reasons.

**Table 1:** Overview of clinical, genetic and imaging findings from eight patients (comprising four families, F1-F4) with ACO2 deficiency.

	F1-P1	F1-P2	F2-P1	F2-P2	F2-P3	F3-P1	F3-P2	F4-P1
Patients								

**Gender**	Female	Male	Female	Female	Female	Female	Female	Female
**Ethnicity**	Arab	Arab	Arab	Arab	Arab	Arab	Arab	Caucasian
**Current age**	10y	4y	12y	11y	6y	17y	14y	† at 46mo
**Genotype**	c.336C>G/ c.336C>G	c.336C>G/ c.336C>G	c.336C>G/ c.336C>G	c.336C>G/ c.336C>G	c.336C>G/ c.336C>G	c.336C>G/ c.336C>G	c.336C>G/ c.336C>G	c.1859G>A/ c.2048G>T
	p.Ser112Arg/ p.Ser112Arg	p.Ser112Arg/ p.Ser112Arg	p.Ser112Arg/ p.Ser112Arg	p.Ser112Arg/ p.Ser112Arg	p.Ser112Arg/ p.Ser112Arg	p.Ser112Arg/ p.Ser112Arg	p.Ser112Arg/ p.Ser112Arg	p.Gly620Asp/
								p.Gly683Val
**Phenotype**								
Hyptonia	+	+	+	+	+	+	+	+
Ataxia	+	+	+	+	+	+	+	+
Optic atrophy	+	+	+	+	+	+	+	+
Strabismus	+	+	+	+	+	+	+	+
Seizures	+	-	+	+	+	-	+	+
Microcephaly	+	+	+	+	+	+	+	+
Intellectual disability	+	+	+	+	+	+	+	+
Sensorineuronal Hearing loss	+	+	na	na	-	+	na	-
Failure to thrive	+	+	+	+	+	+	+	+
**Imaging findings**								
**(Age at MRI)**	4y	12y	7mo	16mo	1y	3y	na	34mo
Cerebral atrophy	+	+	+	+	+	+	na	+
Cerebellar atrophy	+	+	-	-	+	+	na	+
Thin CC	+	-	+	-	-	+	na	+
White matter abnormalities	+	+	-	+	-	+	na	+

na = not available; F1 and F2 have been previously published [2].

### Reagents

All reference compounds, ultra LC-MS grade solvents and reagents for the metabolomics analysis were purchased from Sigma-Aldrich AG (Buchs, Switzerland).

### Genetic studies

**Fig 1:**
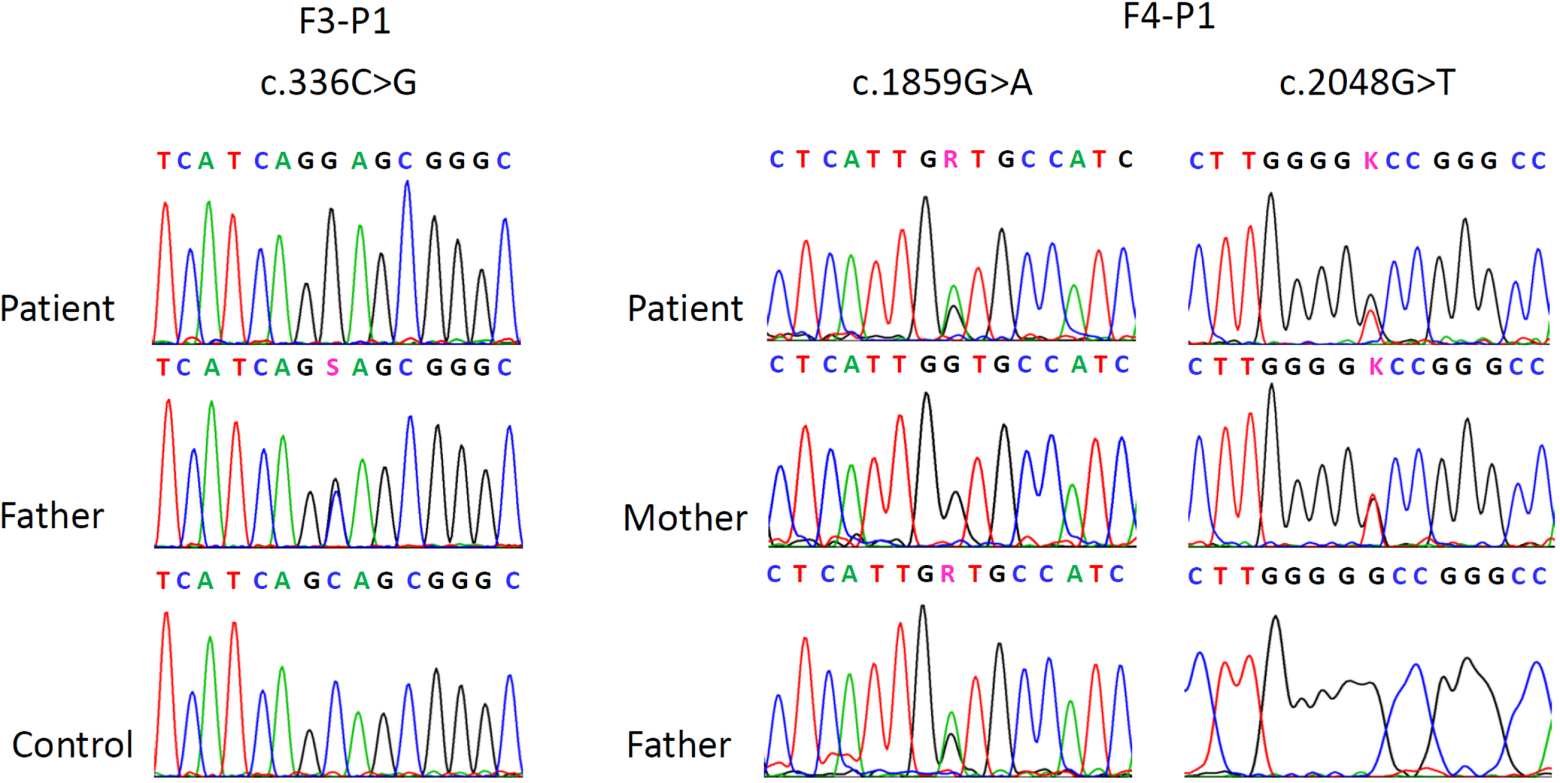
Partial electropherograms from Sanger sequencing showing the mutations in F3-P1 and F4-P1. On the left side, the homozygous mutation in F3-P1, the heterozygous state of the father of F3-P1 and the wildtype in a control individual. On the right side the compound heterozygous mutations of patient F4-P1 with respective heterozygous and wild-type states in the parents.

Five patients (F1-P1/P2; F2-P1/P2/P3) were previously found to harbor the homozygous pathogenic mutation c.336C>G in the *ACO2* gene [2]. Two other siblings residing in the same village also tested positive for this mutation (Fig 1). In brief, genomic DNA was extracted from whole blood using the FlexiGene DNA kit (Qiagen, Hilden, Germany) according to the manufacturer’s instructions. A PCR based assay was used to amplify the regions in which the mutations are located (primer sequences available upon request). Direct sequence analysis of PCR products was performed in both forward and reverse directions using the ABI prism 3130 Genetic Analyzer (Applied-Biosystems, Foster City, CA, USA).

In patient F4-P1 whole exome sequencing was performed using the Agilent SureSelectXT Clinical Research Exome Kit (V5) (Agilent Technologies) with paired-end sequencing (HiSeq SBS Kit v4, 125 Fwd-125 Rev) on a HiSeq2500 System (Illumina Inc.) on genomic DNA derived from peripheral blood. Raw fastQ files were aligned to the hg19 reference genome using NextGene (Softgenetics). Variants observed in at least 16% of reads with sufficient quality level were analyzed for de novo, compound heterozygous and homozygous calls (with Minor Allele Frequency ≤ 2%). The resulting variants were investigated in silico for deleterious effects by SIFT, PhyloPhen, LRT, Mutation Taster, Mutation assessor, FATHMM, GERP and CADD, by associations of the affected gene with epilepsy or intellectual disability and by literature search for evident functional information. The candidate *ACO2* variants were confirmed and tested for segregation by Sanger sequencing.

### Metabolomics data acquisition and preprocessing

All plasma samples were analyzed by liquid chromatography-mass spectrometry (LC-MS; Dionex Ultimate XRS3000 UHPLC coupled to a Q-Exactive high resolution, accurate mass spectrometer (Thermo Scientific, Sunnyvale, CA, USA)). Chromatographic separations were achieved using a 2.1 x 100mm Kinetex HILIC column (Phenomenex, Torrance, CA, USA). Mobile phases used were: A) 50% acetonitrile in 5mM ammonium formate (pH 3.2), and B) 90% acetonitrile in 5mM ammonium formate (pH 3.2). Total run time was 15 minutes with a 10 minute linear gradient running from 100% B to 100% A. Solvents were pumped at 400 μL/minute with a column temperature of 30°C and sample chamber held at 5°C. Mass spectrometric measurements were acquired in positive and negative ionization modes through the heated electro-spray ionization (HESI) source. The untargeted metabolomics data set was acquired with the MS detector in full-scan mode (Full-MS) with data-dependent (dd-MS2) acquisition of fragment ions from the top-5 most abundant ions per scan. Mass spectrometer settings for full-MS were as follows: In-source CID 0.0 eV, μscans = 1, resolution = 70,000, AGC target 1e6, max IT = 35 ms, scan range 67 to 1000 *m/z*. Detector setting for dd-MS2 were: μscans = 1, resolution = 17,500, AGC target 1e5, max IT = 80 ms, loop count = 5, isolation window 4.0 *m/z*, NCE 30.0, intensity threshold 1.3e4, apex trigger 2 to 4s.

Raw data were collected in Xcalibur (Thermo) and converted to.mzXML format using the MSconvert.exe [11]. Data preprocessing was performed in R (x64, v3.1.0) the free software environment for statistical computing and graphics (http://www.r-project.org/). Feature detection, retention time correction and peak grouping were done using XCMS [12, 13]. All peaks of interest were manually inspected (EIC) for peak shape and alignment, and then mined against the MetaCyc (http://metacyc.org/; [14], Metlin (https://metlin.scripps.edu/;[15] Human Metabolome (http://www.hmdb.ca/), and KEGG (http://www.kegg.jp/kegg/) databases. Date of the last database access was March 16, 2016. All metabolic pathway annotations were done in python with mummichog v.1.0.5 [16].

### Statistical analysis

**Fig 2:**
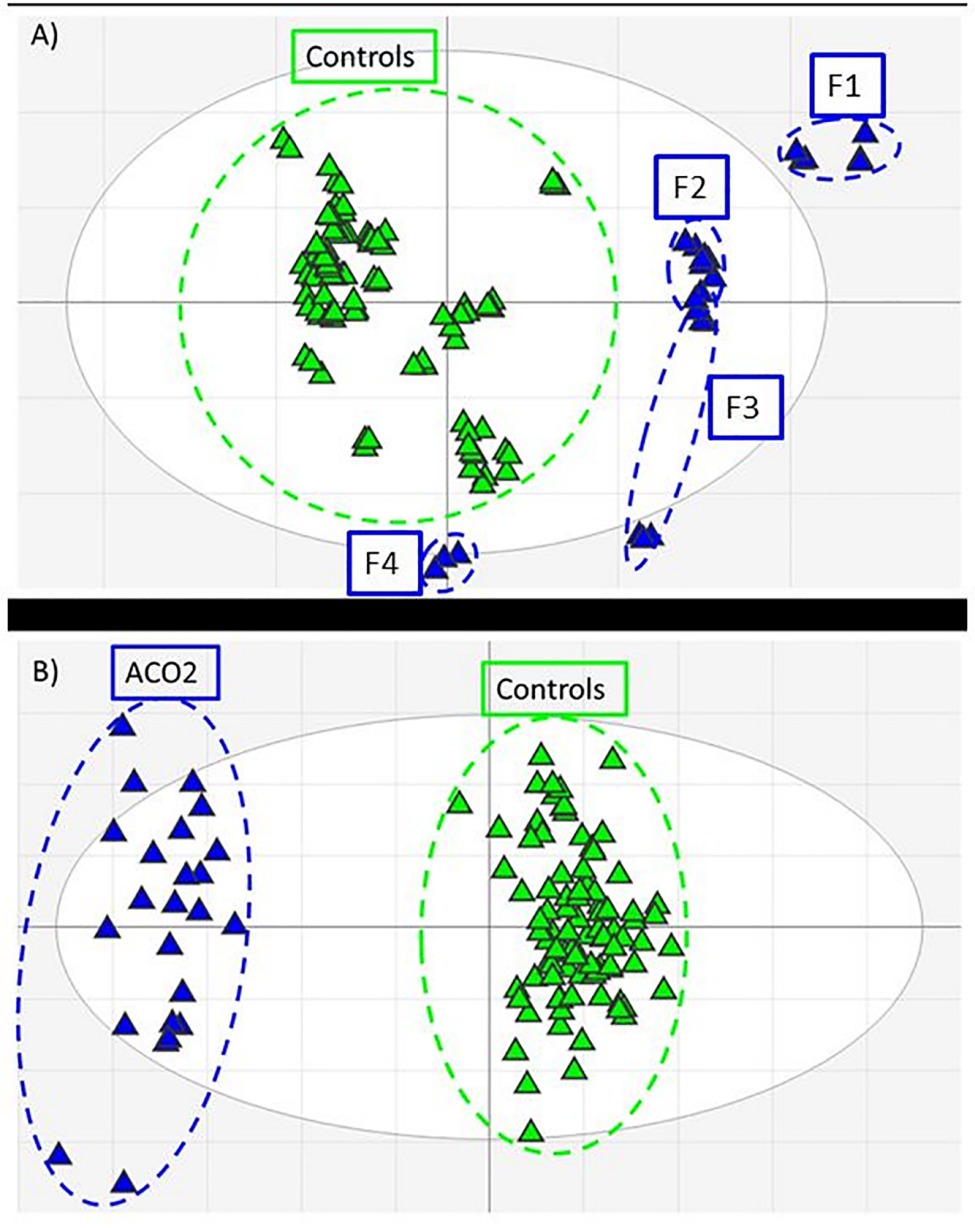
Multivariate analysis score plots of *ACO2*-deficient (blue) and matched control plasma (green) metabolomics data features. A) Principle components analysis (PCA) indicates a clear distinction between *ACO2*-deficient and control profiles. F1-F4 correspond to Table 1. Metabolic variations caused by perturbed metabolic flux due to *ACO2*-deficiency as well as varying exogenous inputs (e.g. diet, medication) result in observed data clustering; B) Supervised orthogonal-partial least squares discriminant analysis (OPLS-DA) with data classification reveals strong feature discrimination between *ACO2*-deficient and control data sets.

Data pretreatment included 1) noise filtering and missing value imputation, where features missing from at least 75% of data were eliminated 2) sum normalization was performed on total intensity data, i.e. the sum of all variables for each sample is calculated and used as normalizing factor for each variable. Each variable is then transformed as a fraction of the total spectral sum. All data scaling, normalization and univariate analysis were performed in R [17]. Multivariate data analysis was performed in SIMCA v13.0.3 (Unimetrics, Malmö, Sweden). Data matrices containing mass over charge (*m/z*), and normalized ion signal intensity were analyzed with the unsupervised principle components analysis (PCA; Fig 2A). Orthogonal projection to a latent structure-discriminant analysis (OPLS-DA; Fig 2B) was used to evaluate and rank the differential metabolite expression profiles. Relative metabolite ratios were calculated by comparing the integrated area under curve for the target metabolite. False-discovery rates were calculated in GraphPad Prism 6 (GraphPad Software Inc.).

## Results

### Genetic analysis

Whole exome sequencing in patient F4-P1 covered about 96.6% of the targeted region with ≥20 independent sequence reads and revealed a total of 3 de novo variants, 9 pairs of compound heterozygous and 2 homozygous variants (≤ 2% Minor Allele Frequency). Taking into account predicted deleterious effects and associations of the affected gene with epilepsy or intellectual disability in these candidate variants revealed two novel compound heterozygous mutations in the gene *ACO2* (NM_001098) [2] (Fig 1). The mutations were located in exon 15 (c.1859G>A, p.Gly620Asp) and 16 (c.2048G>T, p.Gly683Val) and were predicted to be deleterious by 5 of 6 and 2 of 6 prediction tools, respectively (SIFT (Damaging / Tolerated), PolyPhen (Damaging / Benign), LRT (Damaging / Damaging), Mutation Taster (Disease causing / Disease causing), Mutation assessor (Medium / Neutral), FATHMM (Tolerated / Tolerated), CADD score (17 / 14.4).

### Metabolomics analysis

The final negative ion data matrix comprised 758 features with p < 0.05 and a fold-change > 1.5 (up or down) between case and cohort sample sets. PCA of the resultant data matrix revealed differential clustering of the ACO2 cohort against the control cohort with 66% of the variation within the training set explained by the model and 43% of the variation in the training set predicted by the model according to cross validation in the 16^th^ component (R2X (cum) = 0.66; Q2 (cum) = 0.43) (Fig 2A). Application of supervised OPLS-DA allowed a detailed differential expression analysis between the ACO2 and control cohorts (R2X (cum) = 0.41; R2Y (cum) = 0.94; Q2 (cum) = 0.88) (Fig 2B). An overall false-discovery rate (FDR) of 0.01 was calculated by an empirical Bayesian method based on moderated t-statistics [18]. Data mining and feature annotation led us to correlate multiple ions to individual metabolites based on adduct and fragmentation pattern matching. As each of these data features has an individual signal strength (i.e. ion intensity) an average was calculated for all features attributed to each metabolite (Table 2).

**Table 2:** Annotation and average fold-change of data features corresponding to metabolite identifications.

Metabolite	KEGG id	^a^match form	m/z	^b^delta m/z	^c^avg. fold change
cis-Aconitate	C00417	M-H[–]	173.0077	-0.0014	-36.9
		M(C13)-H[–]	174.0111	-0.0014	
		M+Cl[–]	208.9847	-0.0006	
		M+Cl37[–]	210.9818	-0.0005	
Isocitrate	C00311	M-H[–]	191.0184	-0.0013	-17.7
		M+Cl[–]	226.9954	-0.0005	
		M+Cl37[–]	228.9925	-0.0004	
		M+ACN-H[–]	232.0462	-0.0001	
a-Ketoglutarate	C00026	M-H[–]	145.0128	-0.0014	-4.3
		M+CH3COO[–]	205.0342	-0.0006	
		M-CO2+H[1+]	103.0385	-0.0005	
Succinate	C00042	M+Br[–]	196.9458	0.0009	1.1
		M+ Br81[–]	198.9439	0.001	
Fumarate	C00122	M+Cl37[–]	152.9766	-0.0003	-1.4
Malate	C00149	M+ACN-H[–]	174.0394	-0.0014	-1.1
		M+CH3COO[–]	193.0342	-0.0006	
Phosphoenolpyruvate	C00074	M+CH3COO[–]	226.9954	-0.0003	-6.9
		M-HCOOK+H[1+]	85.0282	-0.0002	
		M-CO2+H[1+]	124.9988	-0.0011	
Glutamate	C00302	M-H[–]	148.0423	-0.0008	1.8
		M+HCOO[–]	192.0496	-0.0012	
		M-C3H4O2+H[1+]	76.0393	-0.0001	
		M-HCOOH+H[1+]	102.0544	-0.0007	
		M-CO2+H[1+]	104.0701	-0.0006	
		M-CO+H[1+]	120.0647	-0.0008	
		M-H2O+H[1+]	130.0489	-0.001	
		M+H[1+]	148.0591	-0.0014	
		M+H2O+H[1+]	166.0711	0	
α-,β-hydroxybutyrate	C05984	M-H[–]	105.0371	-0.0001	-21.8
		M+Na-2H[–]	124.0136	-0.0011	
		M+HCOO[–]	149.044	-0.0009	
		M+CH3COO[–]	163.0598	-0.0008	

^a^) adduct or fragment ion matched to metabolite identification

^b^) mass difference between the calculated and observed mass of the matched feature.

^c^) average fold change calculated for all matched forms for each annotated metabolite.

## Metabolic fingerprint for aconitase deficiency

### TCA metabolites

**Fig 3:**
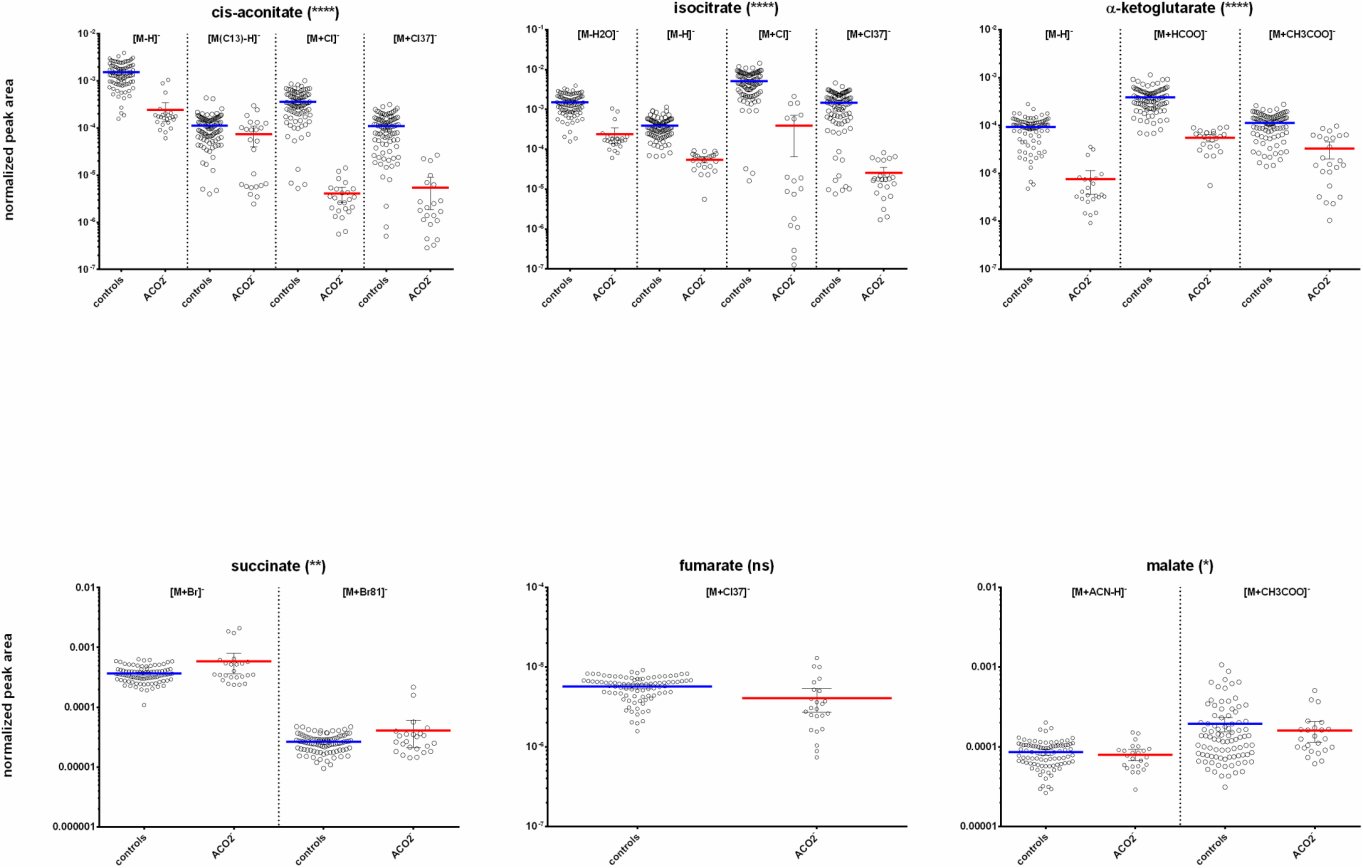
Box-plots indicating differential plasma concentrations of core citric acid cycle metabolites observed between the ACO2 and control cohorts. All observed ion features corresponding to each annotated metabolite are shown for clarity e.g. adducts, isotopes. Asterisk indicate level of statistical significance (**** = p < 0.0001; * = p < 0.01; ns = not significant).

Orthogonal discriminant analysis of the ACO2 vs control negative ionization data matrices provided a ranked list of differentially expressed features between the sample sets. A meta-analysis [19] of comparative data matrices from each family against the control cohort revealed several statistically significant features associated with the citric acid cycle. The series of citric acid cycle metabolites were tentatively identified from the data as being differentially expressed in the ACO2 cohort plasma versus the matched control plasma samples (Fig 3). The features identified in the data as most relevant to a genetic mutation in mitochondrial aconitase were cis-aconitate and isocitrate. However differential levels of other downstream TCA metabolites were also observed, including α-ketoglutarate (AKG). Subsequent targeted analysis of individual ACO2 patient plasma profiles against corresponding age- and sex-matched controls revealed a 17.7-fold relative decrease for isocitrate, a 36.9-fold average relative reduction of cis-aconitate, while α-ketoglutarate was reduced 4.3-fold in the *ACO2* deficient cohort (Fig 3; Table 2). The citric acid cycle metabolites ‘downstream’ from α-ketoglutarate i.e. succinate, malate and fumarate were not dramatically altered.

It should be noted that isocitrate is isobaric with its precursor metabolite, citrate, both having the molecular formula C_6_H_8_O_7_. Due to the co-elution and shared monoisotopic mass of isocitrate and citrate, we turned to their respective MS/MS fragmentation patterns for confirmation. We were able to confirm the presence of lower isocitrate in the study cohort plasma through the analysis of MS/MS fragmentation of reference isocitrate and citrate aligned with the observed daughter ions of isocitrate and citrate (obs. [M-H] *m/z* 173.00755; Metlin *m/z* 173.0078; Δppm -1.4) (S2 Fig).

### Additional metabolites with differential expression in aconitase deficiency

**Fig 4:**
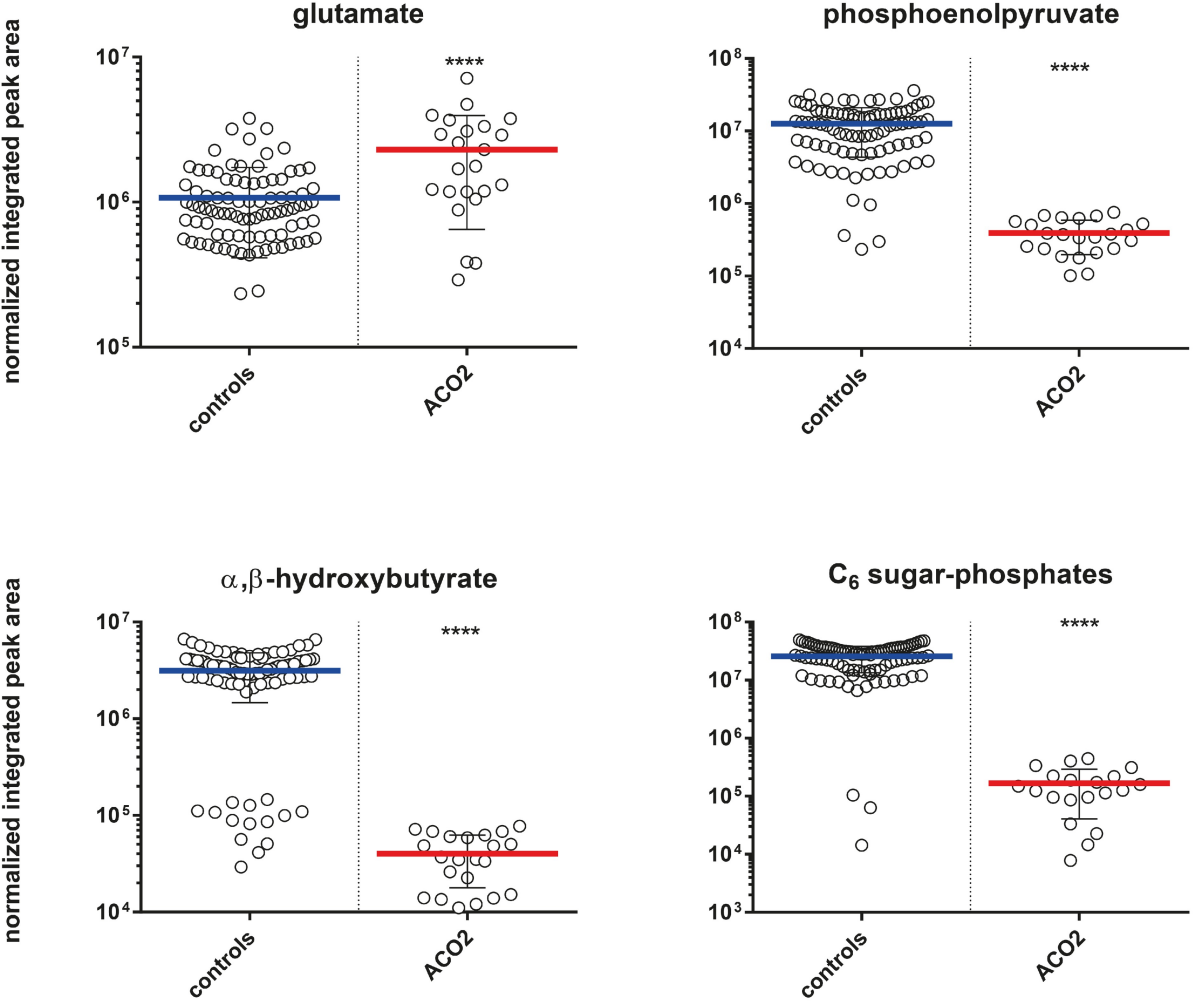
Box-plots indicating differential plasma concentrations of proposed phenotypically relevant metabolites observed between the ACO2 and control cohorts. Asterisks indicate level of statistical significance (**** = p < 0.0001).

Multivariate analysis of the ACO2 plasma and control sample cohorts has revealed the differential expression of various other metabolites which may correspond to the clinical phenotype. For example, plasma levels of glutamate displayed a 1.8-fold relative increase in ACO2 plasma when compared to matched controls. The glycolysis metabolites C_6_ sugar phosphates and phosphoenolpyruvate were downregulated 22.7-fold and 6.9-fold, respectively. α, β-hydroxybutyrate, an end-product of fatty acid beta-oxidation, was downregulated with a 21.8-fold decrease, (Fig 4).

## Discussion

The enzyme aconitase (aconitate hydratase; EC 4.2.1.3) exists as cytosolic (ACO1) and mitochondrial (ACO2) isoforms, both of which catalyze the reversible isomerization of citrate to isocitrate (S1 Fig). The primary function of ACO1 is the regulation and maintenance of iron homeostasis within the cell [20], while ACO2 plays an important role in the regulation of ATP generation [21]. Both isoforms of aconitase contain a covalently bound [4Fe-4S] iron-sulfur cluster, which is highly sensitive to oxidation and required for catalytic activity.

In metabolomics analysis of the *Acon*-knockdown fly study, authors found increased levels of cis-aconitate, citrate/isocitrate ratio and acetyl-CoA, metabolites proximal to the metabolic block, while downstream TCA cycle metabolites including alpha-ketoglutarate (AKG), succinate, fumarate and malate were decreased, albeit a mild perturbation [22]. The authors further identified significantly decreased intermediate metabolites of glycolysis, markedly decreased triacylglyceride levels and decreased ATP levels.

Our plasma metabolomics analysis in eight patients with genetically proven *ACO2* deficiency identified significantly decreased TCA metabolites that are located downstream of the metabolic block. We further found decreased glycolysis and fatty acid breakdown metabolites. Generally these first studies in humans with ACO2 deficiency agree with the findings in the *Acon*-knockdown flies. OXPHOS measurement in a fresh muscle biopsy of an affected patient showed decreased respiratory chain enzyme activity indicating an impairment of oxidative phosphorylation but was normal in two further patients (data not shown). Mitochondrial respiratory chain dysfunction has been identified in a number of neurodegenerative disorders [23–26] but so far there is no clear proof of consistent secondary mitochondrial dysfunction in ACO2 deficiency.

In the following sections, we discuss individual metabolites of the presented metabolic profile that result in the dysfunction of several metabolic energy pathways and provide more insight into the broader metabolic effect of *ACO2* deficiency.

### TCA cycle metabolites and glutamate metabolism

We found significantly decreased cis-aconitate, isocitrate and α-ketoglutarate, metabolites that are found downstream of the metabolic block, while other TCA intermediates including succinate, malate and fumarate were not dramatically altered. These results indicate an impairment of the citric acid cycle though some metabolites seem to be restored by anaplerotic reactions. Alpha-ketoglutarate for example, serves as a major entry and exit point for carbon to and from the Krebs cycle and is essential for the oxidation of fatty acids, amino acids, and glucose [27]. In the TCA cycle, AKG is produced by the oxidative decarboxylation of isocitrate catalyzed by isocitrate dehydrogenase (IDH; S1 Fig). AKG can also be produced anaplerotically from glutamate by oxidative deamination by glutamate dehydrogenase. Our finding of reduced AKG in *ACO2* deficient patients is intriguing as we observe slight elevation in plasma glutamate levels. AKG levels are thus not restored by glutamate deamination. It moreover appears that glutamate metabolism is secondarily affected although the mechanism is not yet clear. In the adult-onset olivopontocerebellar neurodegeneration, decreased AKG levels combined with increased glutamate levels have been associated with a partial deficiency of glutamate dehydrogenase [28].

Neurotoxicity of *ACO2* deficiency may be promoted by both increased glutamate and reduced AKG levels. Glutamate-induced neurotoxicity is a well-established concept that may contribute to brain damage and epileptic activity in *ACO2* deficiency. Metabolomics data showed elevated glutamate levels, although routine analysis of glutamate in serum was normal in most of our patients. However, intracellular increase of glutamate may induce alterations of membrane potentials and ion channels. AKG can directly protect cellular macromolecules against ROS damage during seizure [29]. Reduced AKG levels thus result in an impaired protection from oxidative stress.

### Glycolysis metabolites

Mitochondrial aconitase catalyzes the stereospecific isomerization of citrate to isocitrate. Citrate sits at the crossroad of glycolysis and fatty acid synthesis and as such acts a key regulator of energy production. Citrate exerts a negative feedback on glycolysis through negative regulation of phosphofructokinase 1 and 2 [30, 31]. *Acon*-knockdown flies showed significantly decreased intermediate metabolites of glycolysis, namely glucose-1-phosphate, glucose-6-phosphate, fructose-6-phosphate, glyceraldehyde-3-phosphate, 2-phosphoglycerate, 3-phosphoglycerate, phosphoenolpyruvate (PEP) and pyruvate [22]. In our patient cohort, we could not identify elevated citrate levels, however, citrate may be quickly processed into other associated secondary pathways. We observed markedly decreased C_6_-sugar phosphates and phosphoenolpyruvate indicating impaired glycolysis.

### Metabolites of fatty acid metabolism

Citrate also promotes fatty acid synthesis through allosteric activation of acetyl-CoA carboxylase [32, 33]. Acetyl-CoA carboxylase generates malonyl-CoA, an allosteric inhibitor of carnitine palmitoyltransferase-1 that controls the influx of long chain fatty acids into mitochondria for β-oxidation [34, 35]. Consecutively, we observed significantly decreased α, β-hydroxybutyrate as an indicator of low fatty acid breakdown. This is consistent with Cheng et al., who found markedly decreased triacylglyceride levels in the *Acon-*knock-down flies at day 3 after birth, indicating an activation of fatty acid synthesis for alternative energy storage [22].

### Mitochondrial oxidative phosphorylation

Decreased ACO2 activity results in slowed generation of NADH and FADH_2_ that are required electron carriers for oxygen reduction in the electron transport chain (ETC) coupled with oxidative phosphorylation. Limited electron transport through the ETC consequently decreases ATP production [36] and leads to secondary mitochondrial respiratory chain dysfunction. In the *Acon*-knockdown flies, ATP content was reduced indicating an impairment of downstream oxidative phosphorylation. One single patient (F4-P1) of our cohort had OXPHOS measurement in a fresh muscle biopsy that showed reduced complex I/III enzyme activity, while complex II and II/III showed a borderline enzyme activity (data not shown). Impairment of oxidative phosphorylation leads to increased reactive oxygen species (ROS) that finally result in cell death [37]. Mitochondrial aconitase (ACO2) contains an 4Fe-4S cluster that is particularly sensitive to mitochondrial reactive oxygen species such as superoxide radical O^2-^ [37]. Oxidative inactivation of 4Fe-4S cluster results in loss of an non-ligated iron atom (i.e. 3Fe-4S) and consecutively in formation of H_2_O_2_ radicals and accumulation of Fe^2+^ [37]. Neurotoxicity induced by mitochondrial oxidative stress has been demonstrated for several neurodegenerative disorders. Furthermore, reduced aconitase activity has been found in Huntington disease, progressive supranuclear palsy, Friedreich ataxia [23, 38, 39] and Alzheimer’s disease [40].

## Conclusion

Here we report for the first time a potentially diagnostic plasma metabolic profile for *ACO2* deficiency. The profile includes several metabolites of the TCA cycle and associated metabolic pathways. The complex pathomechanisms of *ACO2* deficiency are not yet completely elucidated. However, investigations in the *Acon*-knockdown fly model and our own metabolomics data indicate that this genetic defect affects three major energy pathways, namely oxidative phosphorylation, glycolysis and fatty acid oxidation. Neurotoxicity may be caused by the accumulation of glutamate, the formation of radical species and the lack of ATP. We suggest that following complete validation of this metabolome profile, plasma be analyzed in patients who present with a combination of hypotonia, intellectual disability, seizures, optic atrophy and cerebellar atrophy on MR images. Herein we demonstrate that metabolome profiling is a powerful tool to characterize disease mechanisms and pathogenicity of mutations.

## Supporting information

S1 Fig The core reactions of the citric acid cycle [1]. Aconitase (aconitate hydratase; EC 4.2.1.3) catalyzes the stereospecific isomerization of citric acid to isocitric acid. The reaction intermediate cis-aconitic acid is indicated in brackets.
(TIF)

S2 Fig Mass spectral fragmentation pattern matching for isocitrate. Fragmentation patterned for the isobaric reference materials (citric and isocitric acid) were compared to the ACO2-deficient plasma MS/MS data.
(TIF)
