# Supplementary figures and images for: Plasma metabolomics reveals a diagnostic metabolic fingerprint for mitochondrial aconitase (ACO2) deficiency
Source: PLoS One. 2017 May 2;12(5):e0176363. doi: 10.1371/journal.pone.0176363 (PMC5413020; doi:10.1371/journal.pone.0176363)

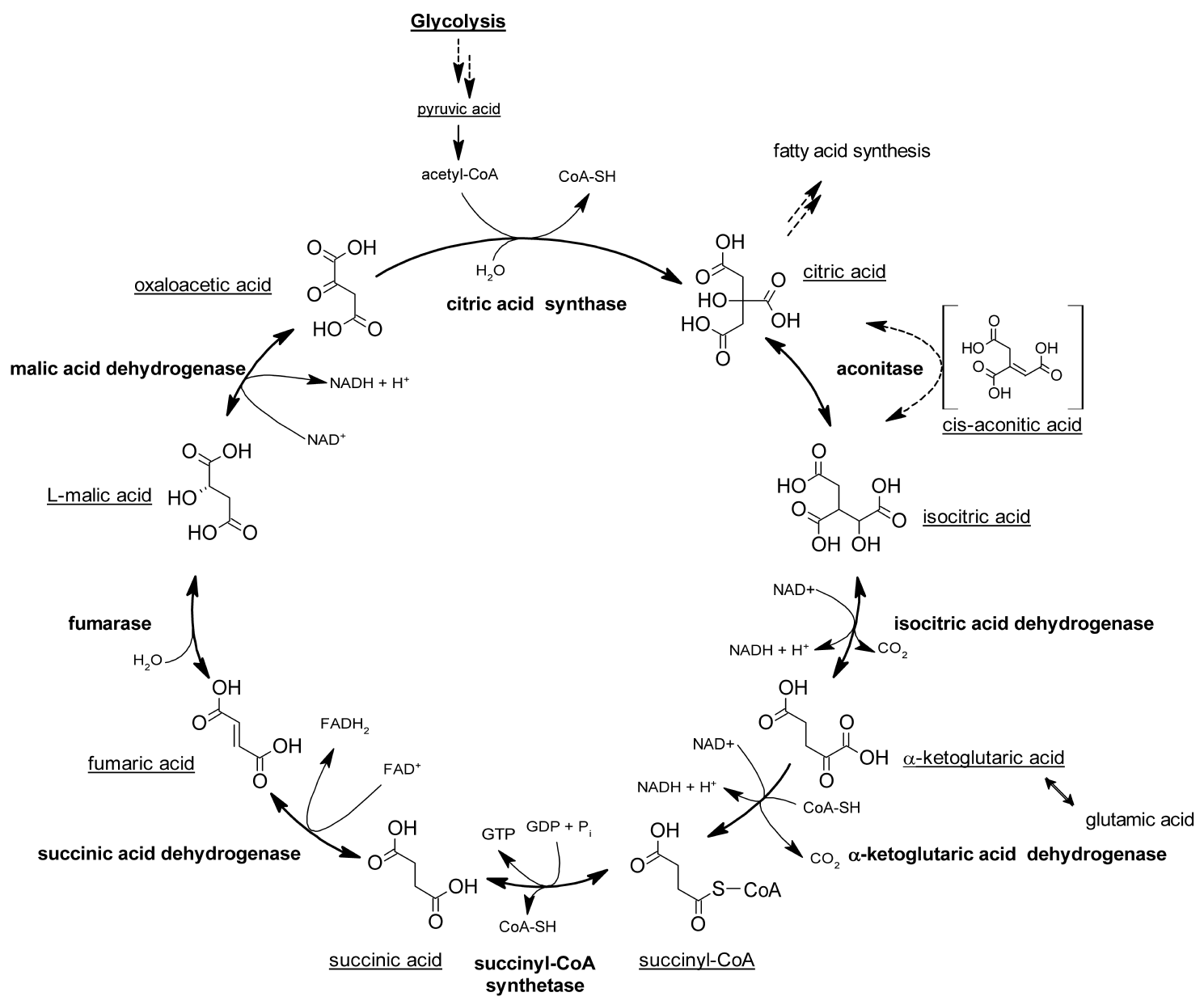

Supplement: S1 Fig — Aconitase (aconitate hydratase; EC 4.2.1.3) catalyzes the stereospecific isomerization of citric acid to isocitric acid. The reaction intermediate cis-aconitic acid is indicated in brackets. (TIF) [file pone.0176363.s001.tif]

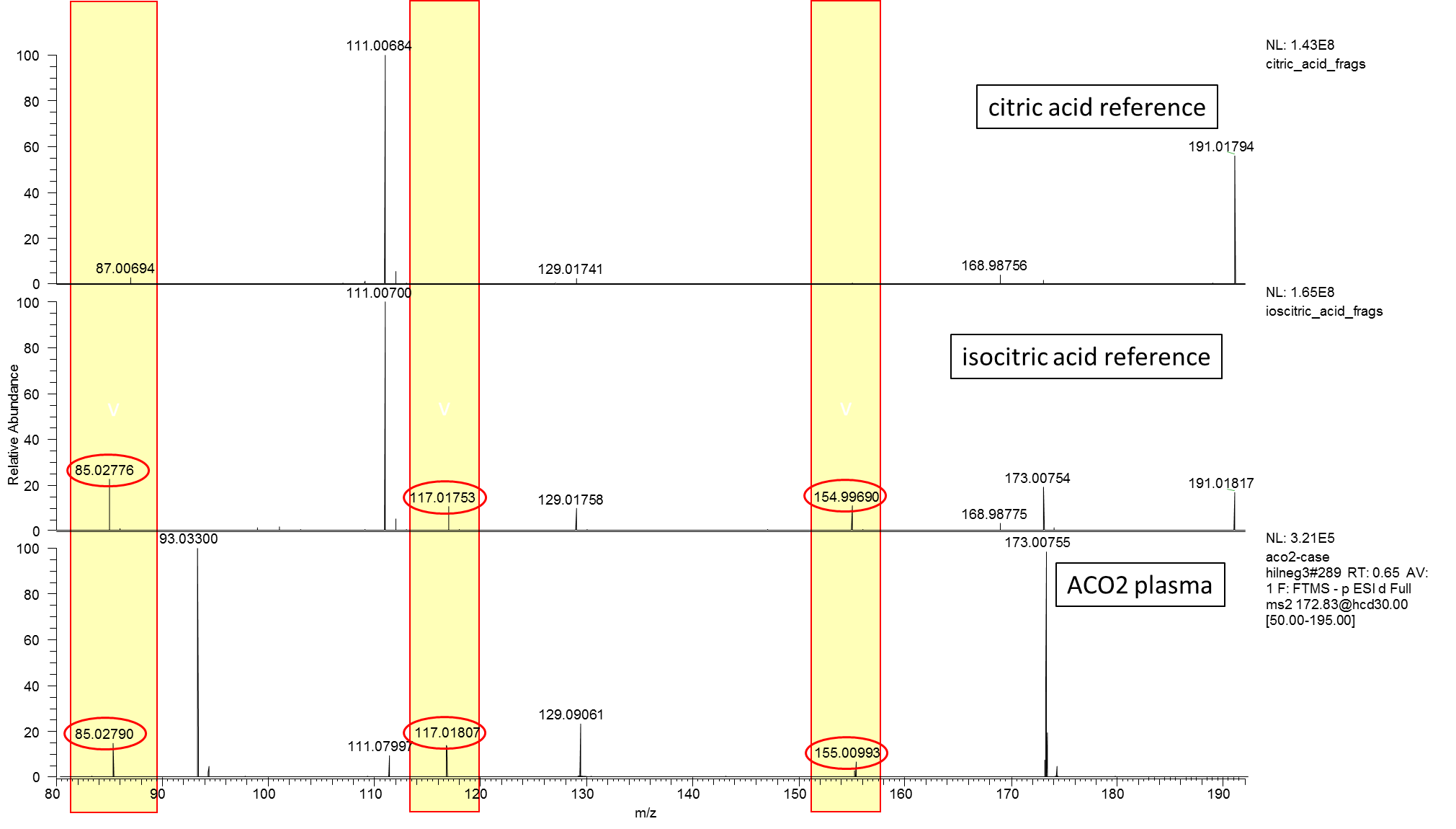

Supplement: S2 Fig — Fragmentation patterned for the isobaric reference materials (citric and isocitric acid) were compared to the ACO2-deficient plasma MS/MS data. (TIF) [file pone.0176363.s002.tif]
